# Osteoclasts control reactivation of dormant myeloma cells by remodelling the endosteal niche

**DOI:** 10.1038/ncomms9983

**Published:** 2015-12-03

**Authors:** Michelle A. Lawson, Michelle M. McDonald, Natasa Kovacic, Weng Hua Khoo, Rachael L. Terry, Jenny Down, Warren Kaplan, Julia Paton-Hough, Clair Fellows, Jessica A. Pettitt, T. Neil Dear, Els Van Valckenborgh, Paul A. Baldock, Michael J. Rogers, Colby L. Eaton, Karin Vanderkerken, Allison R. Pettit, Julian M. W. Quinn, Andrew C. W. Zannettino, Tri Giang Phan, Peter I. Croucher

**Affiliations:** 1 Department of Oncology, University of Sheffield Medical School, University of Sheffield, Beech Hill Road, Sheffield, South Yorkshire S10 2RX, UK; 2 Mellanby Centre for Bone Research, University of Sheffield Medical School, University of Sheffield, Beech Hill Road, Sheffield, South Yorkshire S10 2RX, UK; 3 Garvan Institute of Medical Research, 384 Victoria Street, Sydney, New South Wales 2010, Australia; 4 St Vincent's Clinical School, Faculty of Medicine, UNSW Australia, Sydney, New South Wales 2010, Australia; 5 School of Biotechnology and Biomolecular Sciences, UNSW Australia, Sydney, New South Wales 2010, Australia; 6 South Australian Health and Medical Research Institute, Adelaide, South Australia 5000, Australia; 7 Department of Hematology and Immunology, Vrije Universiteit Brussel, Brussels 1090, Belgium; 8 Department of Human Metabolism and Clinical Biochemistry, University of Sheffield Medical School, University of Sheffield, Beech Hill Road, Sheffield, South Yorkshire S10 2RX, UK; 9 Mater Research Institute, The University of Queensland, Translational Research Institute, 37 Kent Street, Woolloongabba, Queensland 4102, Australia; 10 School of Medical Sciences, University of Adelaide, Frome Road, Adelaide, South Australia 5000, Australia

## Abstract

Multiple myeloma is largely incurable, despite development of therapies that target myeloma cell-intrinsic pathways. Disease relapse is thought to originate from dormant myeloma cells, localized in specialized niches, which resist therapy and repopulate the tumour. However, little is known about the niche, and how it exerts cell-extrinsic control over myeloma cell dormancy and reactivation. In this study, we track individual myeloma cells by intravital imaging as they colonize the endosteal niche, enter a dormant state and subsequently become activated to form colonies. We demonstrate that dormancy is a reversible state that is switched ‘on' by engagement with bone-lining cells or osteoblasts, and switched ‘off' by osteoclasts remodelling the endosteal niche. Dormant myeloma cells are resistant to chemotherapy that targets dividing cells. The demonstration that the endosteal niche is pivotal in controlling myeloma cell dormancy highlights the potential for targeting cell-extrinsic mechanisms to overcome cell-intrinsic drug resistance and prevent disease relapse.

## Abstract (web summary)

Therapy resistant dormant myeloma cells contribute to disease relapse. Here, the authors use intravital microscopy to track the location of these cells and demonstrate that they hone to the endosteal niche within the bone.

## Graphical abstract

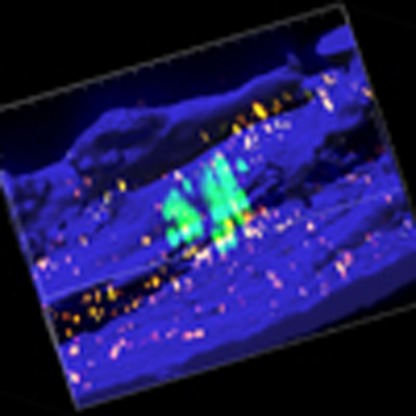

Cancer cell dormancy is a poorly understood and often neglected stage in the evolution of many cancers, where extrinsic signals from the tumour microenvironment suppress active growth and proliferation, until more favourable conditions arise^1^,^2^. This is a major clinical problem, as dormant cancer cells may disseminate at an early stage in the disease^3^, become resistant to conventional therapies that target dividing cells^1^ and persist as minimal residual disease (MRD), which can be reactivated to promote disease relapse long after treatment cessation^4^. In the skeleton, dormant cells may co-exist in equilibrium with the bone microenvironment for years before reactivation and clinical relapse. In this regard, multiple myeloma, a primary haematological malignancy arising in bone, exemplifies the major therapeutic challenges posed by cancer cell dormancy. While novel therapies that selectively target cell ‘intrinsic' cancer properties have improved survival^5^, patients continue to relapse and myeloma remains largely incurable. Hence, understanding the ‘extrinsic' environmental factors that regulate myeloma cell dormancy is required to deliver complementary treatment strategies to overcome drug resistance and achieve complete remission and cure.

Recently, whole-genome sequencing has identified key driver mutations and complex mutation patterns during the natural history of myeloma within individual patients^6^. These longitudinal analyses have revealed marked intra-clonal heterogeneity and shifting clonal dominance during disease progression and in response to drug treatments^7^,^8^,^9^. The ‘waxing and waning' of different myeloma clones (clonal tides) suggests that cancer cell growth and proliferation are not fixed genetic programmes^8^,^9^ that follow a linear model, but rather a branching and parallel, ‘Darwinian', model of clonal evolution that is subject to external selective pressures^6^,^10^. These data suggest that myeloma cell clones are able to reversibly switch ‘on' or ‘off' depending on the presence of favourable or unfavourable environmental signals. A critical component of this tumour microenvironment is the bone niche where myeloma cells initially colonize and are believed to reside^11^. However, the nature of this niche and mechanisms that control myeloma cell occupancy are poorly defined.

Haematopoietic stem cells (HSCs) have long been known to occupy unique niches within the bone marrow microenvironment and this controls HSC dormancy, self-renewal and mobilization. These specialized microenvironments contain cells of the osteogenic lineage, perivascular cells and/or endothelial cells, and remodelling of these niches by osteoclasts regulates niche occupancy^12^,^13^,^14^,^15^,^16^,^17^,^18^. More recently, leukaemic cells and other cancer cells, including prostate cancer cells, have been shown to engraft in the HSC niche^19^,^20^,^21^. However, despite the importance of the niche in controlling tumour cell engraftment, the dynamic interactions between colonizing cancer cells and components of these specialized niches, and the impact of these interactions on the long-term fate of these cells, is poorly understood.

Studying the dynamic interactions between dormant myeloma cells and the bone niche is particularly challenging, because it requires high-resolution deep-tissue imaging through intact bone in a live animal. Nevertheless, intravital microscopy of the bone marrow space beneath the bregma in the calvarium^22^ has been successfully used to study haematopoiesis^23^ and the HSC niche^24^,^25^,^26^,^27^, and has recently been applied to study the colonization of bone by leukaemic and myeloma cells^20^,^28^. However, to date, non-destructive microscopic imaging over periods of weeks to months is yet to be performed to longitudinally track the fate of the same individual myeloma cells as they become activated and escape dormancy. This would be a significant advance on longitudinal imaging by bioluminescence, which lacks the image resolution and potential for simultaneous visualization of the cells and structures that make up the bone niche. Furthermore, it would have distinct advantages over cross-sectional intravital microscopy, which may not capture the heterogeneity within and between different animals. Nevertheless, to image dormant myeloma cells *in vivo*, a robust method is required to discriminate resting from actively dividing cancer cells. Intravital dyes such as carboxyfluorescein succinimidyl ester, which label intracellular proteins, have relatively short half-lives and are unsuitable for the study of long-term cancer cell dormancy. Fluorescent stem cell markers have been used to track HSCs and study the epithelial stem cell niche, but these reporters may lack specificity when used in isolation^29^,^30^,^31^,^32^.

To address this, here we have developed a method for dynamic longitudinal imaging of dormant myeloma cells within intact long bones of mice using intravital two-photon microscopy. Myeloma cells are double-labelled with both fixed genetic reporters and lipophilic membrane dyes, which are retained by non-dividing myeloma cells. Intravital microscopy is used to resolve single tumour cells as they arrive in bone and engage in the bone niche, to track the transition from dormancy to active growth, and to follow the serial growth of tumour colonies by repeated imaging of the same mouse at weekly intervals. These studies show that myeloma cells can be held in a dormant state by osteoblast-like cells in the endosteal niche, and this state confers dormant cells with drug resistance. Furthermore, direct activation of osteoclasts can remodel the endosteal surface to release dormant myeloma cells from the niche and stochastically facilitate their reactivation to repopulate the tumour. Thus, myeloma cell dormancy is a reversible state controlled by the extrinsic bone microenvironment, which can be manipulated to change the course of the disease.

## Results

### Colonizing myeloma cells engage in the endosteal niche

**Figure 1:**
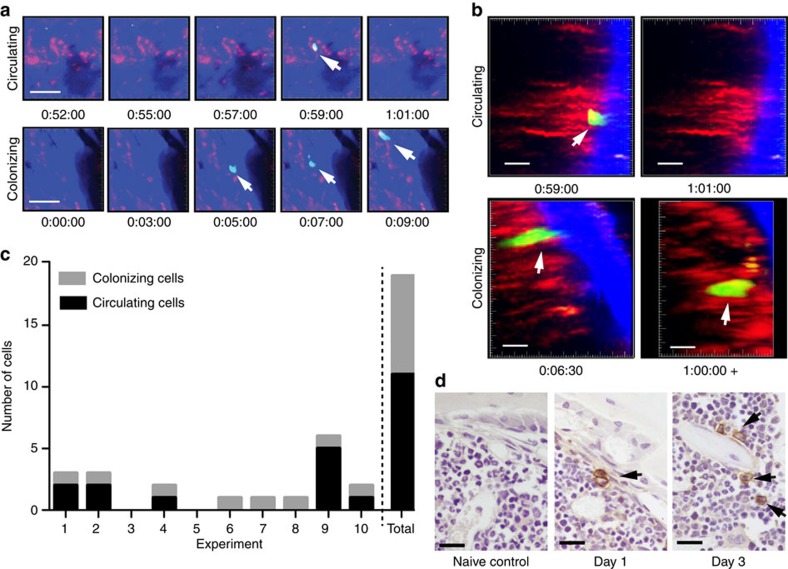
Intravital two-photon microscopy of cancer cells colonizing bone. (**a**) Still-frames capturing the arrival of a circulating tumour cell (top panels) and colonizing tumour cell (bottom panels) in real time. Blue, bone; red, vasculature; green, 5TGM1-GFP myeloma cells. Scale bars, 50 μm. It corresponds to Supplementary Movies 1 and 2. (**b**) Still-frames of the same cells in **a** showing egress of circulating cell from bone after 2 min and retention of a colonizing cell >1 h in the bone. Blue, bone; red, vasculature; green, 5TGM1-GFP myeloma cells. Scale bars, 20 μm. (**c**) Proportion of cells that circulated through or colonized the bone marrow in each experiment and total of all experiments. (**d**) CD138-stained sections from naive, or cell-injected mice at 1 and 3 days post injection. Single CD138^+^ cells (brown, black arrows), haematoxylin counterstain. Scale bars, 20 μm. Data represent at least three experiments.

C57BL/KaLwRijHsd mice were injected with syngeneic 5TGM1-enhanced green fluorescent protein (eGFP) murine myeloma cells and GFP^+^ cells arriving in the intact tibia were visualized in real time by intravital two-photon microscopy (Fig. 1a–c; Supplementary Movie 1). Rare colonizing myeloma cells were observed as they migrated from the marrow space towards endocortical bone surfaces, where they arrested (Fig. 1a–c). Histological analysis confirmed the presence of individual CD138^+^ cells adjacent to endosteal bone surfaces 1 and 3 days after tumour cell inoculation (Fig. 1d). The majority of myeloma cells continued to circulate through the bone marrow, where they were only briefly captured in the imaging volume as they flowed through in blood vessels (Fig. 1a–c; Supplementary Movie 2). These circulating cells were seen throughout the duration of the imaging period (>60 min), although the absolute numbers were likely underestimated, as images were captured at 30-s intervals (Fig. 1c). Nevertheless, real-time intravital imaging demonstrated that individual cancer cells directly seed the endosteal bone niche in the tibia.

### Visualization of subpopulations of myeloma cells *in vivo*

**Figure 2:**
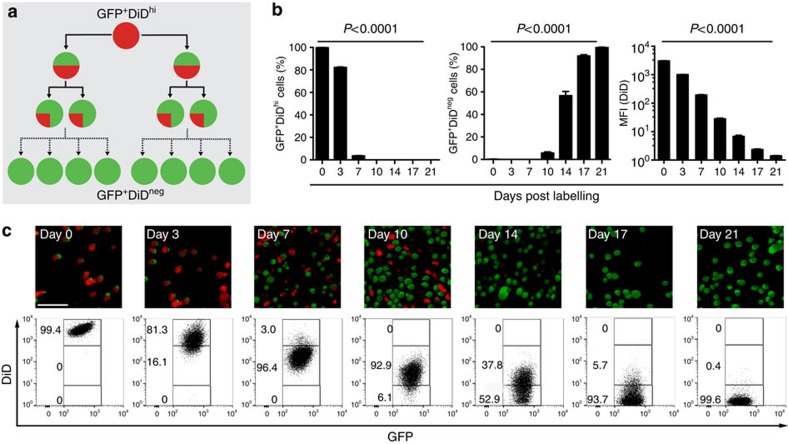
Labelling and tracking dormant cancer cells *in vitro*. (**a**) Schematic of DiD labelling to identify non-dividing cells. (**b**) FACS analysis of the DiD level over 21 days of *in vitro* culture showing percentage of DiD^hi^ and DiD^neg^ cells (left, middle panel) and mean fluorescence intensity (MFI) of the DiD level (mean±s.e.m., one-way ANOVA). (**c**) Fluorescent microscopy (top panels) and FACS analysis (bottom panels) of cultured DiD-labelled cells. Numbers indicate percentages of GFP+ cells in the gates. Scale bar, 150 μm.

**Figure 3:**
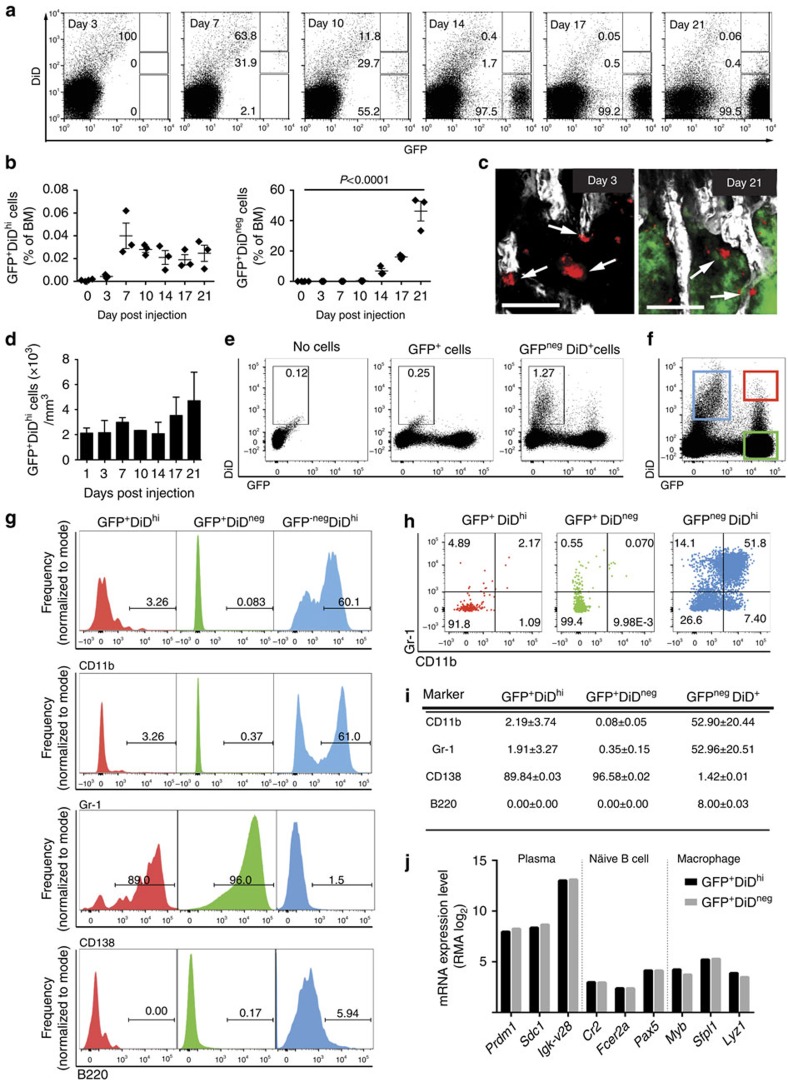
Labelling and tracking dormant myeloma cells *in vivo*. (**a**) FACS analysis of bone marrow (BM) cells after inoculation of GFP^+^DiD-labelled 5TGM1-eGFP cells. Numbers indicate percentages in the gates. (**b**) Percentage of total BM of GFP^+^DiD^hi^ dormant cells and GFP^+^DiD^neg^ cells by FACS analysis from **a** (individual data points and mean±s.e.m., one-way ANOVA). (**c**) Two-photon images of the metaphyseal region of explanted tibias harvested at days 3 and 21. White, bone; red, GFP^+^DiD^+^ cells (white arrows); green, GFP^+^DiD^neg^ cells. Scale bars, 200 μm. (**d**) Enumeration of GFP^+^DiD^+^ cells (mean+s.e.m.). Data represent 4–5 individual mice. (**e**) FACS plots of BM samples from naive (no cells), mice injected with GFP cells not labelled with DiD (GFP^+^DiD^neg^) or injected with DiD-labelled GFP^+^ cells (GFP^+^DiD^+^), % of BM cells presented. (**f**) FACS plot demonstrating the three populations of cells examined in **g**–**i**. (**g**) Histograms showing the frequency of cells that are CD11b^+^, Gr-1^+^, CD138^+^ or B220^+^ for each cell population from **f**. (**h**) FACS plots showing distribution of CD11b and Gr-1-expressing cells in each GFP/DiD population from **f**. (**i**) Table showing the % of cells (mean+s.e.m.) that are CD11b^+^, Gr-1^+^, CD138^+^ or B220^+^ for each cell population from **f**. (**j**) Transcript profile of GFP^+^DiD^hi^ and GFP^+^DiD^neg^ cells showing the expression level of plasma cell, naive B-cell and myeloid lineage genes.

To investigate the heterogeneity in cell growth and proliferation within populations of myeloma cells, we labelled 5TGM1-eGFP cells with 1,1'-dioctadecyl-3,3,3',3'-tetramethylindodicarbocyanine (DiD) (Fig. 2a). *In vitro* cultures of double-labelled cells showed a serial dilution of the dye mean fluorescence intensity as GFP^+^DiD^hi^ cells divided and shared the label among daughter cells, which progressively became GFP^+^DiD^lo^ and finally GFP^+^DiD^neg^ (Fig. 2b,c). Next, mice were injected with double-labelled (GFP^+^DiD^hi^) cells, and the tibia was analysed by two-photon microscopy and fluorescence-activated cell sorting (FACS) analysis. Initially, only GFP^+^DiD^hi^ cells were present (Fig. 3a,b); however, as cells engrafted and divided, the expansion of GFP^+^ cells was associated with a dilution of the DiD label (Fig. 3a,b). At later time points when myeloma burden was high, GFP^+^DiD^hi^ cells, which had retained the label, and therefore had not divided, were still detectable (Fig. 3a,b). Interestingly, the proportion of GFP^+^DiD^hi^ cells appeared higher at day 7 and subsequent days, when compared with earlier time points (Fig. 3b), suggesting that labelled cells can recirculate for several days before eventually colonizing the bone marrow. Two-photon imaging of the entire explanted tibia detected non-divided GFP^+^DiD^+^ cells at all time points, even among developing GFP^+^DiD^neg^ tumours (Fig. 3c,d; Supplementary Fig. 1; Supplementary Movie 3). Furthermore, the numbers of GFP^+^DiD^+^ cells did not change with time, confirming the long-term persistence of these cells (Fig. 3d). DiD labelling was also seen in GFP^neg^ endogenous cells (Fig. 3e,f), which likely reflects membrane transfer^33^, and shows the importance of using a double-labelling approach with a fixed genetic reporter, such as GFP, to unequivocally identify engrafted tumour cells. Flow cytometric analysis demonstrated that >70% of the GFP^−^DiD^+^ cells were Gr-1^+^ and/or CD11b^+^ myeloid cells. The GFP^+^DiD^hi^ population contained only 3.26% Gr-1^+^ and 3.26% CD11b^+^ expressing cells (Fig. 3g–i). Thus, the GFP^+^DiD^hi^ population was highly enriched for GFP^+^DiD^hi^ cells and contained <7% contaminating Gr-1^+^ and/or CD11b^+^ cells. Furthermore, 89% of dormant GFP^+^DiD^hi^ and 96% of the GFP^+^DiD^neg^ cells were CD138^+^, with no difference in intensity between the two populations. Less than 2% of the GFP^neg^DiD^+^ population were CD138^+^ and fewer than 6% were B220^+^ (Fig. 3g,i). B220 was not expressed on GFP^+^DiD^hi^ or GFP^+^DiD^neg^ cells (Fig. 3g,i). Consistent with this, sorted GFP^+^DiD^hi^ cells expressed genes typical of plasma cells, but not naive B cells or myeloid cells (Fig. 3j).

### A subpopulation of myeloma cells is dormant *in vivo*

**Figure 4:**
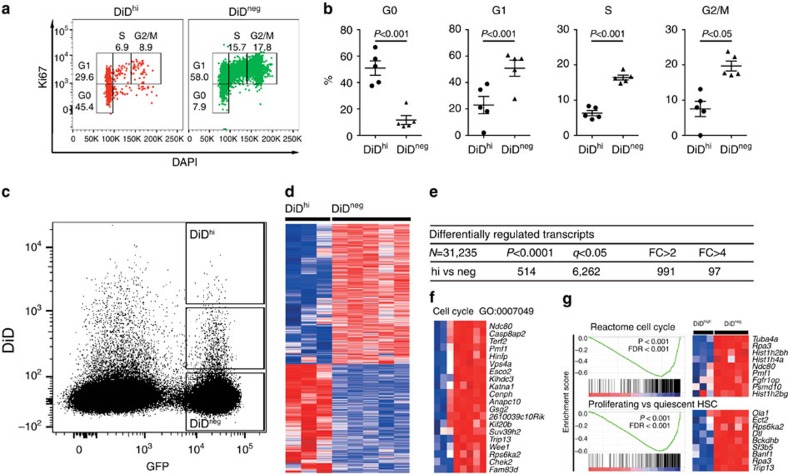
Confirmation of GFP^+^DiD^hi^ cell population as dormant myeloma cells. (**a**) FACS plot showing gating of GFP^+^ DiD^hi^ and GFP^+^ DiD^neg^ cells in G0, G1, S and G2/M cell cycle phases as determined by Ki67 expression and DAPI staining on day 21. FACS plot shown is pooled data from five mice. (**b**) Distribution of DiD^hi^ and DiD^neg^ cells in G0, G1, S and G2/M phase of the cell cycle from gating shown in **a**. Data represent 4–5 individual mice (mean±s.e.m., paired *t*-test). (**c**) Gates used for FACS sorting of GFP^+^DiD^hi^ and GFP^+^DiD^neg^ cells. (**d**) Heatmap showing differentially expressed genes. (**e**) Table of differentially transcribed genes between the three cell populations. (**f**) Gene expression analysis showing genes involved in cell cycle and replication, transcriptional and translational activity, which were expressed at lower levels in dormant cells when compared with GFP^+^DiD^neg^ cells isolated from the same mice. (**g**) Selected regulatory gene sets determined by GSEA, including negative enrichment for gene sets related to cell cycle progression and regulation of HSC quiescence in DiD^hi^ vs DiD^neg^ cells. Dot plots represent concatenated myeloma populations from six tumour-bearing mice.

The long-term retention of DiD in a population of cells suggested that these cells were dormant, whereas those that had lost DiD had undergone membrane dye dilution due to cell proliferation. Significantly more GFP^+^DiD^hi^ cells than GFP^+^DiD^neg^ cells were detected in the G_0_ phase of the cell cycle, as determined by Ki67 and 4,6-diamidino-2-phenylindole (DAPI) staining (*P*<0.001—paired *t*-test; Fig. 4a,b), while significantly less GFP^+^DiD^hi^ cells were in G_1_, S and G_2_/M phases (*P*<0.001, *P*<0.001 and *P*<0.05 paired *t*-test, respectively, Fig. 4a,b). FACS sorting and transcriptional profiling of GFP^+^DiD^hi^ and GFP^+^DiD^neg^ cells from disease-bearing mice revealed two distinct transcriptional profiles with 6,262 differentially expressed genes between the two activation states (Fig. 4c–e). Genes involved in cell cycle and cell replication were downregulated in the GFP^+^DiD^hi^ population when compared with the GFP^+^DiD^neg^ population (Fig. 4f; Supplementary Table 1). Furthermore, gene set enrichment analysis (GSEA)^34^ identified gene sets inversely related to cell cycle, and quiescent versus proliferating HSC signatures (Fig. 4g). Thus, FACS analysis and molecular profiling identified GFP^+^DiD^hi^ cells as a dormant population that has undergone limited numbers of cell divisions as a result of the downregulation of genes that control cell cycle.

Importantly, gene expression analysis revealed that the dormant GFP^+^DiD^hi^ population had a distinct profile of differentially expressed genes when compared with activated GFP^+^DiD^neg^ cells (Fig. 4d). The 100 most significantly upregulated genes and 100 most significantly downregulated genes in GFP^+^DiD^hi^ cells are shown in Supplementary Table 2. GFP^+^DiD^hi^ cells were found to highly express genes related to immune responses, including those encoding interferon-induced molecules and regulatory factors, which have been implicated in controlling breast cancer bone metastasis^35^. Notably, GFP^+^DiD^hi^ cells also expressed higher levels of the cell adhesion molecule *Vcam1*, which has also been implicated in breast cancer metastasis^36^, and *Axl*, a member of the TAM family of receptor tyrosine kinases, implicated in prostate cancer cell dormancy^37^.

### The endosteal niche reversibly controls myeloma cell growth

**Figure 5:**
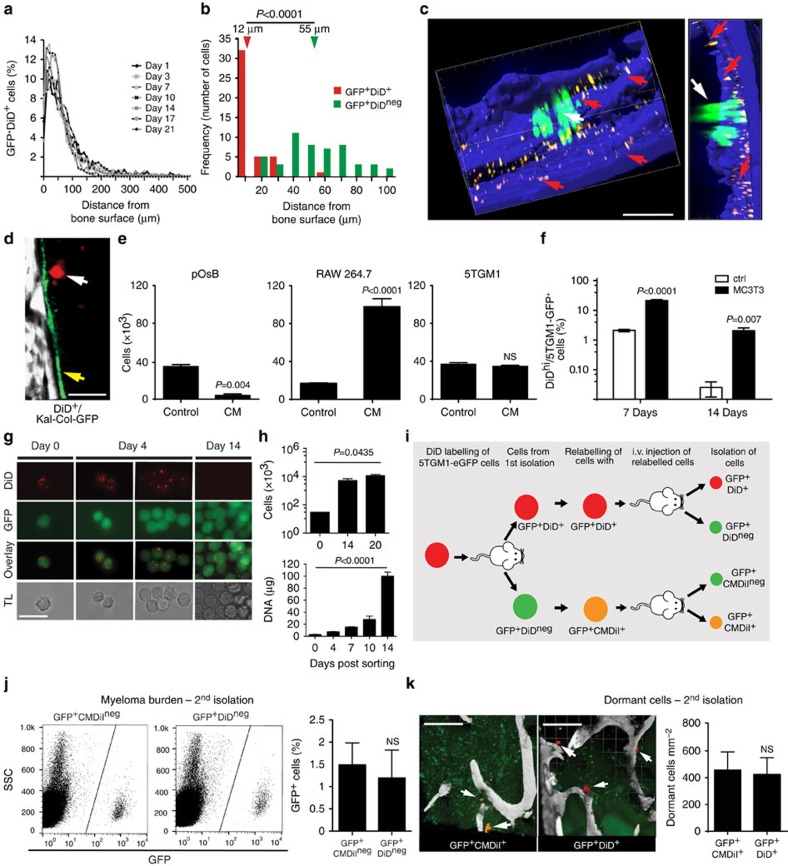
Dormancy can be switched on and off by the bone environment. (**a**) Measurement of distance of GFP^+^DiD^+^ cells to the nearest bone surface. (**b**) Comparison of the location of GFP^+^DiD^+^ and GFP^+^DiD^neg^ cells. Arrows indicate median distance from bone surface. (**c**) Three-dimensional two-photon image of green GFP^+^DiD^neg^ cells (white arrow) and yellow GFP^+^DiD^+^ cells (red arrows) localized to the bone surface, blue; scale bar, 100 μm. (**d**) Two-photon image of a DiD-labelled 5T33MM cell (arrow) localizing to the bone surface (SHG, white) lined with osteoblasts (col2.3 GFP^+^, green). (**e**) Effect of conditioned media (CM) from primary osteoblasts (pOsB, left panel), macrophage cell line (RAW264.7, middle panel) and myeloma cells (5TGM1, right panel) on cancer cell dormancy, unpaired *t*-test. (**f**) Effect of co-culture with osteoblast cell line MC3T3 on cancer cell dormancy. (**g**) Fluorescent and bright-field microscopy from *in vitro* cultures of FACS-sorted GFP^+^DiD^hi^ long-term dormant cells; scale bar, 25 μm. (**h**) Cell count and DNA content of *in vitro* cultures from **g**, one-way ANOVA. (**i**) Schematic for isolation (day 21 post inoculation) and reinoculation of dormant GFP^+^DiD^+^ and activated GFP^+^DiD^neg^ cells. (**j**) FACS analysis of bone from mice after second passage for myeloma cell burden. Data show percentage of GFP^+^ per bone marrow cells (mean±s.e.m., unpaired *t*-test). (**k**) Localization of dormant GFP^+^CMDiI^+^ (white arrows, left panel) and dormant GFP^+^DiD^+^ (white arrows, middle panel) cells adjacent to bone surfaces from two-photon microscopy of *ex vivo* tibia (bone, white) and enumeration of GFP^+^CMDiI^+^ and GFP^+^DiD^+^ cells per mm^2^ bone. Scale bars, 200 μm. Data in **e**,**f**,**h**,**j** and **k** show mean±s.e.m. Data represent at least two experiments. NS, not significant.

We next determined the micro-anatomical location of GFP^+^DiD^hi^ and GFP^+^DiD^neg^ cells by two-photon microscopy. These studies revealed that dormant GFP^+^DiD^hi^ cells were preferentially located in direct contact with endosteal bone surfaces (defined by the second-harmonic signal from bone collagen), and this position did not change over time (Fig. 5a–c; Supplementary Movie 3). In contrast, activated GFP^+^DiD^neg^ cells were preferentially found at locations distant from the bone surface (Fig. 5b,c). To confirm engagement of the endosteal bone niche by dormant myeloma cells, we inoculated DiD-labelled 5T33MM cells (GFP^neg^ cells) into Kal-Col-GFP transgenic mice that express the emerald GFP protein (emdGFP) under the control of the type-I collagen 2.3-kb promoter, which is expressed by osteoblastic lineage cells^38^. Individual DiD^hi^ 5T33MM cells were localized directly adjacent to bone surfaces occupied by emdGFP^+^ cells (Fig. 5d). These data suggested that the endosteal niche actively suppressed myeloma cell growth. We therefore cultured DiD^hi^ cells in osteoblast-conditioned media or co-cultured them with MC3T3 murine osteoblast-like cells and demonstrated increased numbers of GFP^+^DiD^hi^ cells in both systems as compared with control. These data suggest that factors made by osteoblasts suppressed proliferation, maintaining GFP^+^DiD^hi^ cells in a dormant state (Fig. 5e,f). In contrast, media conditioned by RAW264.7 macrophages, a surrogate for osteoclasts, promoted the proliferation and growth of GFP^+^DiD^hi^ cells, whereas myeloma cell-conditioned media had no effect (Fig. 5e).

We next asked whether release of dormant myeloma cells from the endosteal niche would reverse this suppression, and whether re-engagement with the endosteal niche by active myeloma cells would return them to a dormant state. In the first instance, we FACS-sorted GFP^+^DiD^hi^ cells from disease-bearing mice on day 17 and cultured them *in vitro*. By day 4, cultured individual GFP^+^DiD^hi^ cells had divided to form small clusters of 2–6 GFP^+^ cells with a diminished DiD label (Fig. 5g). By day 14, the cells had completely lost the DiD label through proliferation, and the cell number and DNA content had correspondingly increased (Fig. 5g,h). This was recapitulated *in vivo* by FACS-sorting GFP^+^DiD^hi^ cells, 21 days after cell inoculation, and relabelling with DiD before secondary inoculation into naive mice (Fig. 5i). Upon transfer, these previously dormant cells proliferated and formed tumours. Reciprocally, FACS-sorted active GFP^+^DiD^neg^ cells, labelled with an alternative membrane dye CMDiI, were also able to recolonize bone and form tumour (Fig. 5j). Importantly, a small fraction of the CMDil-labelled cells localized adjacent to the endosteal bone surface, retained their dye label and hence did not divide (Fig. 5k). Thus, even though the cells had previously been actively dividing, re-engagement with the endosteal niche was able to suppress the growth of a subpopulation of these cells and switch on dormancy *in vivo.* Remarkably, dormant cells were detected adjacent to bone surfaces in mice inoculated with either GFP^+^DiD^hi^- or GFP^+^DiD^neg^-relabelled cells at similar frequencies, indicating that dormancy was not an intrinsic property of the cell (Fig. 5k). Collectively, these data suggest that suppression of myeloma cell growth by the endosteal niche is a transient reversible state that can be switched ‘on' or ‘off'.

### Limited numbers of dormant myeloma cells get activated

**Figure 6:**
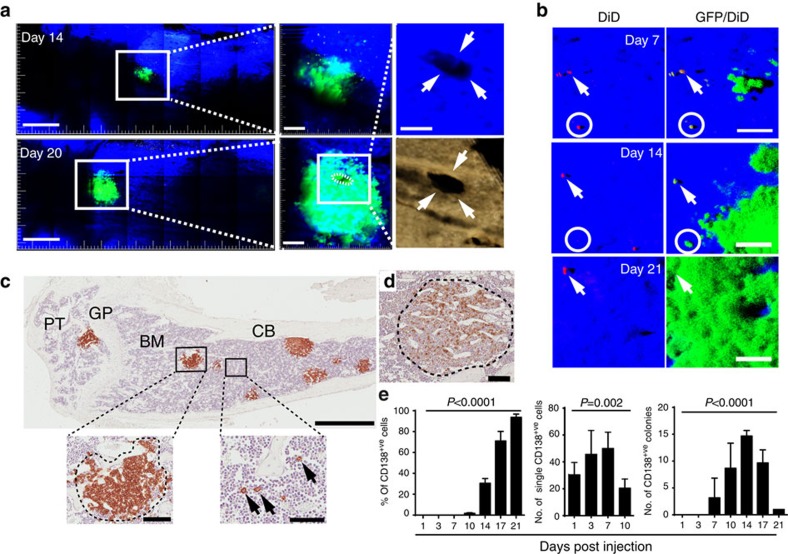
Longitudinal intravital two-photon imaging of dormant cancer cell activation and colony formation. (**a**) Mosaic tile maximum intensity projection of the same 3 mm region of interest in the same tibia over time at 14 and 20 days showing expansion of a single GFP^+^DiD^neg^ colony (green); scale bar 400 μm. Middle panel enface *z*-stacks of each colony, right top, higher magnification of the MIP image from the 20-day *z*-stack with the green removed revealing a hole in the cortical bone. This hole was associated with the GFP^+^DiD^neg^ colony at 20 days and was also captured by 3D reconstruction of a MicroCT scan, right bottom; scale bars, 40 μm. (**b**) Three-dimensional maximum intensity projection of the same tibia at 7, 14 and 21 days showing expansion of a GFP^+^DiD^neg^ colony (green), persistence of the dormant GFP^+^DiD^+^ cell (white arrow) and disappearance of a transitioning GFP^+^DiD^+^ cell (white circle). Scale bars, 50 μm. (**c**) Immunohistochemistry (IHC) showing eight individual CD138^+^ cancer cell colonies (brown) within the bone marrow on day 14 (left panel). Scale bar, 1 mm. Inset shows an individual colony (left: scale bar, 100 μm) and multiple single CD138^+^ cells (black arrows) adjacent to bone surfaces (right: scale bar, 50 μm). BM, bone marrow; CB, cortical bone; GP, growth plate; PT, proximal tibia. (**d**) IHC of CD138^+^ stained cancer cell colony from the 5T2MM murine model of myeloma (scale bar, 100 μm). (**e**) Quantification of IHC-stained CD138^+^ single cells and colonies within the bone marrow. Data are represented per section of the entire bone. Data (mean±s.e.m., one-way ANOVA) represent at least two experiments.

To study dormant myeloma cells and their transition to actively proliferating cells, we performed longitudinal two-photon imaging of the tibia from the same mice at 7, 14 and 20–21 days post cell inoculation (Fig. 6a,b). At day 14, small GFP^+^ colonies were visualized adjacent to bone surfaces (Fig. 6a), which on re-imaging at day 20 had increased in size and were associated with what appeared to be osteolytic lesions in the cortical bone (Fig. 6a, right panels; Supplementary Movie 4). At day 7, individual dormant GFP^+^DiD^hi^ cells were localized to endocortical bone surfaces and limited numbers of small individual colonies, containing <10 GFP^+^DiD^neg^ cells per colony, were identified (Fig. 6b). Re-imaging at days 14 and 21 demonstrated the persistence of dormant GFP^+^DiD^hi^ cells (arrows) and the development of activated GFP^+^DiD^neg^ colonies (Fig. 6b). Importantly, dormant GFP^+^DiD^hi^ cells, transitioning to actively growing GFP^+^DiD^neg^ cells, were also visualized (Fig. 6b, circles). Immunohistochemistry revealed discrete CD138^+^ tumour colonies (Fig. 6c) and single, CD138^+^ cells that had not divided adjacent to bone surfaces, consistent with the intravital imaging (Fig. 6c, bottom right panel). Similar results were obtained with the 5T2MM murine model of myeloma (Fig. 6d). The proportion of medullary cavity occupied by CD138^+^ cells increased until it was completely replaced by tumour (Fig. 6e, left panel). Notably, the tumour colony count reached a maximum at day 14 post inoculation before decreasing in number, reflecting the coalescence of colonies (Fig. 6e, right panel). In contrast, the number of single CD138^+^ cells always exceeded the maximum number of colonies (Fig. 6e, middle panel). Taken together, these data suggest that only a small proportion of GFP^+^DiD^hi^ cells are activated to form proliferating GFP^+^DiD^neg^ colonies, while the remainder were maintained in a dormant state.

### Dormant myeloma cells are resistant to melphalan

**Figure 7:**
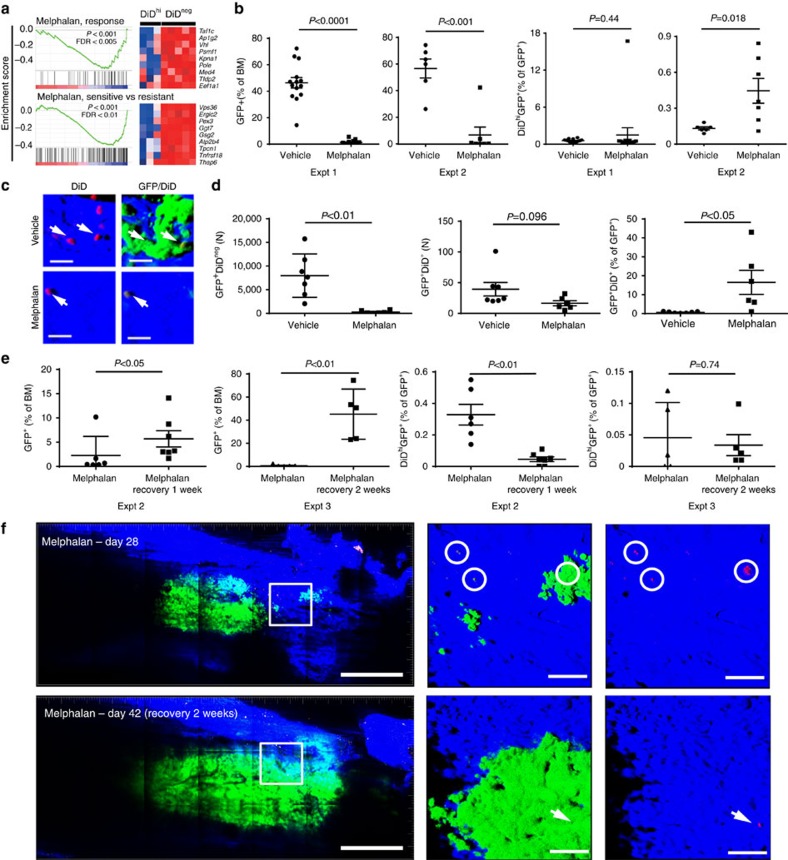
Preclinical testing of drug efficacy against dormant cancer cells. (**a**) Gene set enrichment analysis of genes associated with melphalan sensitivity downregulated in the GFP^+^DiD^hi^ as compared with GFP^+^DiD^neg^ cells. (**b**) FACS analysis of total GFP^+^ cells (left panels. % of bone marrow cells) and GFP^+^DiD^hi^ cells (right panels, % of GFP^+^ cells) for two separate experiments (expt 1 and expt 2). Data show mean±s.e.m. and represent 6–10 mice per group. (**c**) Three-dimensional maximum intensity projection from *ex vivo* two-photon imaging of bone from vehicle (top panels) and melphalan-treated mice (bottom panels). Scale bar, 20 μm. (**d**) Data from Imaris generated spots on mosaic tiles of femurs from vehicle and melphalan-treated mice for number of GFP^+^ cells (left panel) and number of GFP^+^DiD^+^ cells (middle panel) per bone. Percent GFP^+^DiD^+^ of GFP^+^ cells is also shown (right panel). Data show mean±s.e.m. and represent six mice per group (**e**) FACS analysis of GFP^+^ cells (left panels, % of bone marrow cells) and GFP^+^DiD^hi^ cells (right panels, % of GFP^+^ cells) in melphalan-treated mice to day 28 (melphalan) or recovered from melphalan (melphalan recovery: 1 or 2 weeks) for two separate experiments (expt 2 and expt 3). Data show mean±s.e.m. and represent 6–10 mice per group, unpaired *t*-test. (**f**) Mosaic tiled images of intravital two-photon imaging of the same mouse at days 28 when melphalan was ceased, and day 42 after 2 weeks of recovery following melphalan cessation. Middle panel, higher-magnification images of the region in the white box in the same position of the bone at days 28 and 42. At day 28, small green (GFP^+^DiD^neg^) colonies and single red (GFP^+^DiD^+^) cells (white circles) are shown more clearly when the green channel is removed from the image (right panels). At day 42, all of the red (GFP^+^DiD^+^) cells have been activated and the green (GFP^+^DiD^neg^) colonies expanded, except for a single red cell (arrow). Scale bars, 500 μm (left panel), 100 μm (middle and right panels).

Since conventional anticancer drugs target actively dividing cells, we examined the response of GFP^+^DiD^hi^ and GFP^+^DiD^neg^ cells to melphalan, an alkylating agent commonly used to treat myeloma. Indeed, microarray analysis and GSEA demonstrated that gene sets involved in the response and sensitivity to melphalan were negatively enriched in dormant GFP^+^DiD^hi^ cells, suggesting that these cells may be resistant to melphalan due to their lack of proliferation (Fig. 7a). To test this, we treated disease-bearing mice with melphalan and quantified the response by enumeration of dormant GFP^+^DiD^hi^ cells and activated GFP^+^DiD^neg^ cells by FACS analysis and two-photon microscopy of explanted tibia (Fig. 7b–d). While all mice responded to melphalan with significant decreases in tumour burden, the response was variable with some mice achieving >99% reduction, and others only able to achieve 90–99% reduction (Fig. 7b). Nevertheless, despite the decrease in GFP^+^ myeloma cells, dormant GFP^+^DiD^hi^ cells were remarkably stable and could be detected in all mice while on treatment, with the proportion of GFP^+^DiD^hi^ cells increasing relative to total GFP^+^ cells (Fig. 7b). Consistent with this, two-photon microscopy demonstrated a reduction in GFP^+^DiD^neg^ cells and a persistence of GFP^+^DiD^hi^ cells apposed to endosteal surfaces (Fig. 7c,d). These data suggest that dormant GFP^+^DiD^hi^ cells are resistant to melphalan and may contribute to disease relapse upon drug withdrawal. In contrast, treatment of disease-bearing mice with bortezomib, which targets both dividing and non-dividing cells, reduced myeloma burden and also the proportion of GFP^+^DiD^hi^ dormant cells, suggesting that dormant cells may respond differently to different agents (Supplementary Fig. 2).

To directly address the contribution of GFP^+^DiD^hi^ cells to disease relapse, we treated disease-bearing mice with melphalan for 14 days, identified individual dormant GFP^+^DiD^hi^ cells and actively growing GFP^+^DiD^neg^ colonies by intravital imaging and then re-imaged the same mice either 7 or 14 days after treatment withdrawal to track their fate. Following treatment cessation, FACS analysis demonstrated a significant increase in the number of GFP^+^DiD^neg^ cells, confirming escape from therapeutic control and mimicking disease relapse (Fig. 7e). However, unlike in the treatment setting, dormant GFP^+^DiD^hi^ cell numbers decreased following treatment withdrawal, consistent with their reactivation and their role in repopulating the tumour (Fig. 7e). In support of this, longitudinal intravital two-photon microscopy demonstrated a reduction in individual GFP^+^DiD^hi^ cells as the tumour repopulated the marrow following cessation of treatment at day 28 (Fig. 7f, circles).

### Bone resorption activates dormant myeloma cells

**Figure 8:**
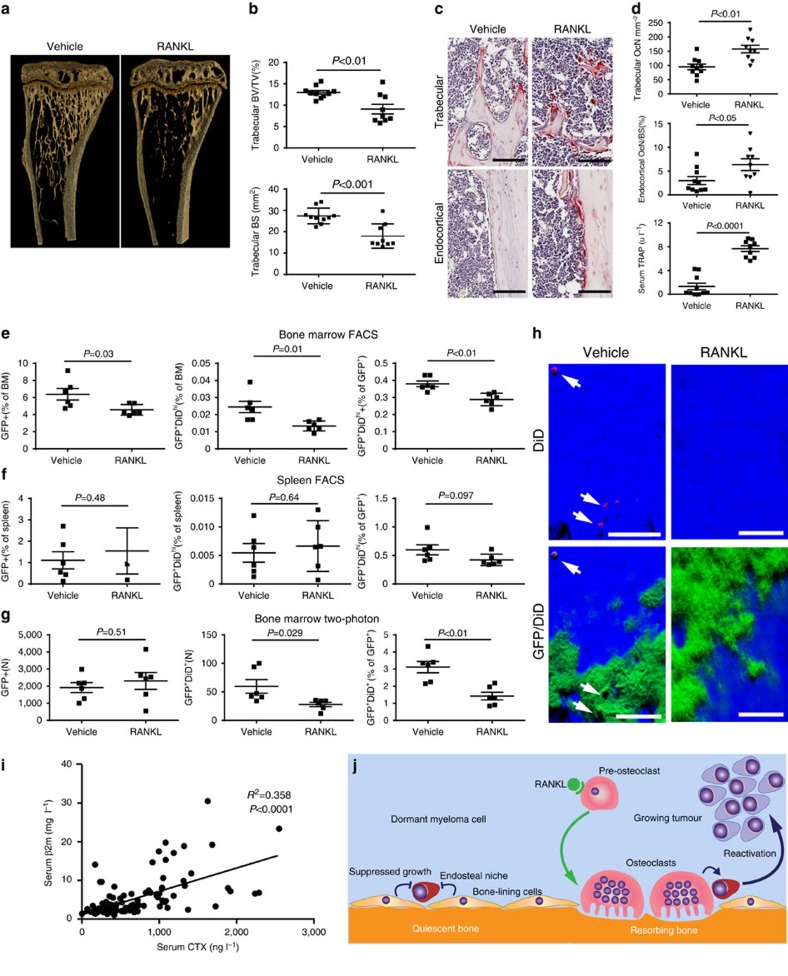
RANKL-driven alterations in the endosteal niche activate dormant tumour cells. (**a**) Three-dimensional reconstructions of MicroCT scans of representative tibia from vehicle- and RANKL-treated naive mice after 3 days of treatment. (**b**) Trabecular bone volume of total volume (BV/TV) and trabecular bone surface (BS) in metaphyseal bone in vehicle- and RANKL-treated mice. (**c**) Representative sections of TRAP-stained tibia from trabecular and endocortical regions of vehicle- and RANKL-treated naive mice; scale bar, 100 μm. (**d**) Histograms of osteoclast surface per mm bone surface for trabecular (top) and endocortical (middle) bone and serum TRAP levels (bottom). Data are from one experiment with 9–10 mice per group. (**e**) FACS analysis of GFP^+^DiD^neg^ cells (left, % of bone marrow cells), GFP^+^DiD^hi^ (middle % of bone marrow cells) and percent GFP^+^DiD^hi^ (right % of GFP^+^) from bone marrow samples. (**f**) FACS analysis of GFP^+^ cells (left, % of spleen cells), GFP^+^DiD^hi^ (middle, % of spleen cells) and GFP^+^DiD^hi^ (right, % of GFP^+^) from spleen samples. (**g**) Analysis of two-photon mosaic tiles of total number of GFP^+^ cells (left), number of GFP^+^DiD^hi^ cells (middle) and GFP^+^DiD^hi^ (right % of GFP^+^) in the analysed endocortical region. Data show mean±s.e.m. and represent 6–10 mice per group, unpaired *t*-test. (**h**) Two-photon generated 3D images of multiple GFP^+^DiD^+^ dormant cells (white arrows) in the vehicle-treated mice compared with few or none in the RANKL treated; scale bars, 60 μm. (**i**) Correlation between serum CTX and serum β2m levels in myeloma patients, Pearson's correlation. (**j**) Schematic depicting myeloma cell dormancy on quiescent bone surfaces and myeloma cell reactivation on actively resorbing bone surface. Data are from one experiment with six mice per group.

To directly test the impact of manipulating the bone microenvironment on disease activity, we treated disease-bearing mice with a soluble form of the ligand for the receptor activator of NFκB (sRANKL), which is expressed by osteoblasts and osteocytes and is critical for osteoclast formation and bone resorption^39^. Daily treatment with sRANKL for 3 days in naive BKAL mice resulted in a significant reduction in trabecular bone volume and bone surface (Fig. 8a,b), along with a significant decrease in trabecular number and an increase in trabecular separation (data not shown). These structural changes in bone were associated with an increase in osteoclasts within trabecular bone regions and on endocortical bone surfaces (Fig. 8c,d) and an increase in serum tartrate-resistant acid phosphatase, a marker of osteoclast activity (Fig. 8d, bottom panel). Importantly, FACS analysis demonstrated that manipulation of the bone microenvironment resulted in a significant decrease in the number of dormant GFP^+^DiD^hi^ cells in the bone marrow, as anticipated (Fig. 8e). Interestingly, although there was also a modest reduction in myeloma burden, there were fewer GFP^+^DiD^hi^ cells as a proportion of the GFP^+^ tumour burden (Fig. 8e). Importantly, sRANKL treatment had no effect on myeloma cells in the spleen (Fig. 8f), confirming that the effect on GFP^+^DiD^hi^ cells was a myeloma cell-extrinsic effect mediated by osteoclasts in the bone, with osteoclast activation associated with the release of GFP^+^DiD^hi^ cells from the endosteal niche. Quantitative assessment of GFP^+^DiD^+^ and GFP^+^DiD^neg^ cells by two-photon microscopy confirmed the decrease in numbers of GFP^+^DiD^+^ cells relative to both bone marrow volume and GFP^+^DiD^neg^ tumour (Fig. 8g,h). Interestingly, intravital imaging did not demonstrate a decrease in GFP^+^DiD^neg^ tumour burden, which may reflect the smaller sample volume or a differential distribution of tumour, compared with analysis by FACS. Thus, sRANKL induces osteoclastic bone resorption and can release dormant myeloma cells from osteoblastic or bone-lining-cell suppression, making them available to contribute to disease progression and/or relapse. To establish whether bone resorption is associated with myeloma burden, we examined serum levels of C-terminal telopeptide (CTX), a biochemical marker of bone resorption, and β2-microglobulin (β2m) as a measure of tumour burden in 118 patients with newly diagnosed myeloma. CTX was significantly correlated with β2m (*R*^2^=0.3588, *P*<0.0001, Pearson's correlation), suggesting that increased bone resorption is related to tumour burden in patients (Fig. 8i). Taken together, these data suggest that dormant cells held in the endosteal niche can be reactivated by osteoclasts remodelling the endosteal niche (Fig. 8j).

## Discussion

Cancer cell dormancy is increasingly recognized as an important clinical problem. Despite improved outcomes from new, targeted therapies, patients continue to suffer from relapse and metastases, which are major causes of morbidity and mortality. Thus, there is an increasing need to understand cancer cell dormancy to prevent drug resistance and disease relapse. This is particularly true in multiple myeloma where patients now achieve complete responses to state-of-the-art combination therapy, yet still harbour drug-resistant dormant myeloma cells as MRD and go on to progress at a later time point. Consequently, understanding the extrinsic control of dormant myeloma cells by the bone microenvironment has the potential to uncover novel therapeutic approaches. These may be used in combination with current treatments to either completely eradicate myeloma cells, or to maintain MRD in a dormant state and achieve long-term remission. Accordingly, we developed a unique, non-destructive, method to track the fate of individual myeloma cells, longitudinally, as they colonize the endosteal niche and form tumour colonies over weeks or months, in live mice by intravital two-photon microscopy. By imaging through a natural optical window in the tibia, we have overcome the long-held misconception that non-destructive intravital microscopy of bone can only be performed in the calvaria. Importantly, the tibia has different developmental origins from the calvaria and may also contain distinct anatomical and functional compartments that constitute the specialized microenvironments required for myeloma cell colonization, retention of dormancy and growth. Using this novel approach, we demonstrated that myeloma cells entered the bone marrow, left the vasculature and migrated directly towards endosteal surfaces where they arrested in sites containing type-I collagen-expressing osteoblasts or bone-lining cells. This is consistent with the immunohistochemical studies reporting the presence of leukaemic cells and primary myeloma cells adjacent to bone surfaces^11^,^40^.

Our intravital studies of myeloma cell dormancy were facilitated by the use of a lipophilic membrane dye, DiD, to identify dormant GFP-expressing myeloma cells that had not undergone cell division. This was supported by the detection of stable numbers of these cells throughout the duration of the experimental time course following initial equilibration, indicating that they are long-lived dormant cells. In addition, analysis of Ki67 expression and DAPI staining showed that the majority of GFP^+^DiD^hi^ cells were in the resting G_0_ phase when compared with the GFP^+^DiD^neg^ population. Furthermore, transcriptional analysis demonstrated that the GFP^+^DiD^hi^ population maintained this long-lived persistent dormant phenotype by downregulating genes that control the cell cycle. Interestingly, a limited number of the GFP^+^DiD^hi^ cells were also Ki67 positive, suggesting that, in certain niches, dormant cells may have recently been activated to divide. This challenges the notion that dormancy is a synchronized period of inactivity and supports the idea that dormancy may instead result from the dynamic interplay between myeloma cells in the multiple heterogeneous niches and may exist in different states of activation.

To establish whether dormancy was a fixed or reversible state, we removed dormant GFP^+^DiD^hi^ cells from their bone niche, relabelled them with DiD and reinoculated them into naive mice. We showed that some of the GFP^+^DiD^hi^ cells remained dormant while others were released from dormancy in their new microenvironment and became activated to form GFP^+^DiD^neg^ colonies. Remarkably, in the reciprocal experiment, when we removed proliferating GFP^+^DiD^neg^ cells and labelled them with CMDiL before reinoculation, these cells not only formed colonies but could also be induced to enter a dormant state in their naive host bone environment. These observations infer that cancer cell dormancy is a reversible state that can be switched ‘on' or ‘off' by microenvironmental signals. Interestingly, we also showed that of the many dormant tumour cells that locate to the endosteal niche, only limited numbers become reactivated to form overt myeloma colonies. This is reminiscent of the ‘clonal tides' and altering clonal dominance that is observed in patients exposed to different drug regimens^6^,^8^,^9^,^10^. In addition, the plasticity of the 5TGM1 cells under these experimental conditions and the fact that 89% of GFP^+^DiD^hi^ cells are CD138^+^ suggests that dormant myeloma cells are distinct from cancer stem cells, which are thought to be poorly differentiated cancer cell precursors^41^, and the myeloma stem cell, which have been reported to be CD138^neg^ (refs 42, 43).

Crucially, the proportion of dormant GFP^+^DiD^hi^ and proliferating GFP^+^DiD^neg^ myeloma cells that form colonies or became dormant after secondary transplantation were comparable. This indicated that stochastic engagement with the relevant bone niche was more important than an intrinsic developmental programme in determining the fate of the transplanted myeloma cells. Consistent with this, osteoblast-conditioned media and co-culture with MC3T3 osteoblast-like cells maintained dormancy and suppressed proliferation, whereas osteoclast-like cell-conditioned media stimulated myeloma cell growth. This is consistent with studies *in vitro*, which show that growth of patient-derived multiple myeloma cells is suppressed when cultured with patient-derived osteoblasts and inhibition of myeloma growth when patient-derived MSCs are delivered systemically or directly into bones of SCID-hu MM-bearing mice^44^,^45^. While the mechanisms responsible for suppression of multiple myeloma cell growth and holding MM cells in a dormant state are poorly defined, they may include release of bone morphogenic proteins^46^ or secretion of Wnt proteins and Wnt antagonists^47^,^48^. Furthermore, our gene expression analysis has identified a number of cellular adhesion molecules and membrane receptors, including *Vcam1* and *Axl*, that could also play a role in interactions between dormant myeloma cells and cells in the endosteal niche. Importantly, expression of these molecules may be used as biomarkers to help identify dormant myeloma cells in bone and may also provide new therapeutic targets to aid in the erradication of these cells. Taken together, these data suggest that the extrinsic bone microenvironment, and likely an endosteal niche, plays a critical role in controlling myeloma cell dormancy.

Dormant GFP^+^DiD^hi^ cells were more resistant than proliferating GFP^+^DiD^neg^ cells to melphalan treatment, but not to the proteasome inhibitor bortezomib. This may argue that dormant cells are intrinsically resistant to chemotherapeutic agents that target dividing cells by virtue of their dormant state. Moreover, even in mice with >99% reduction in tumour burden, GFP^+^DiD^hi^ cells were still detectable and contributed to the re-establishment of myeloma growth following melphalan treatment withdrawal. This ‘awakening' of dormant cells during recovery may be a result of alterations in the local environment. Therefore, we tested the hypothesis that direct manipulation of the bone microenvironment itself would impact on dormancy and provide a mechanism for reactivating dormant cells. Short-term treatment with sRANKL increased osteoclast numbers, resorption activity and decreased the bone surface containing endosteal niches. This osteoclastic activity released GFP^+^DiD^hi^ cells from dormancy and resulted in a specific reduction of GFP^+^DiD^hi^ cells in the bone, but not in the spleen. Consistent with this, serum CTX levels were found to correlate with serum β2m, a marker of tumour burden, in a large cohort of patients with newly diagnosed multiple myeloma. These data suggest that bone resorption, simply by remodelling the endosteal niche as part of normal physiological function, may inadvertently activate dormant tumour cells, arguing that activation of dormant cells is an environmentally controlled stochastic event. Interestingly, despite the increased osteoclast activity following RANKL treatment, not all of the bone surface undergoes resorption and as a result dormant cells are still present, suggesting mobilization events are a localized phenomena. The reason why mobilization of dormant cells was associated with a reduction in myeloma burden is unclear. This could reflect a difference in the rate of growth of mobilized cells following treatment or alternatively that, dormant cells that were mobilized by sRANKL died without undergoing cell division; however, the latter is not consistent with our studies, showing these cells have the capacity to grow *in vitro* and *in vivo* (Fig. 5). Nevertheless, these data suggest that stimulation of bone resorption may result in synchronized activation of dormant cells, thereby rendering them more susceptible to targeted therapies. Or that inhibition of bone resorption by anti-resorptive agents, such as bisphosphonates, may enforce cellular dormancy and prevent myeloma cell activation and relapse.

These data therefore have significant clinical implications. We already know inhibitors of bone resorption, including bisphosphonates and strategies to block RANKL, reduce myeloma burden in experimental models^49^,^50^,^51^. Furthermore, bisphosphonate treatment is associated with improvements in survival that may be independent of the development of skeletal-related events^52^; however, the mechanisms responsible remain unclear. These data may provide at least one explanation for these observations, that is, that bisphosphonate treatment may prevent reactivation of dormant myeloma cells. It could also argue that consideration needs to be given to very early anti-resorptive treatment to prevent activation of dormant tumour cells to sustain long-term remission. This is certainly supported by studies in patients with monoclonal gammopathy of undetermined significance, which show that patients with increased bone resorption are more likely to develop overt myeloma^53^.

The experimental data presented here provide the basis for developing novel approaches to prevent disease progression in myeloma. This could include repurposing bone-active drugs as frontline therapies, to be used in combination with targeted therapies, to prevent the reactivation of dormant myeloma cells and sustain long-term remission. Alternatively, the promotion of bone resorption to release tumour cells from dormancy may facilitate their targeting by the existing armamentarium of drugs to eliminate MRD and achieve long-term remission or even cure. Finally, further studies of the molecular mechanisms that regulate dormancy within the endosteal niche may reveal druggable targets that control myeloma reactivation. Thus, this new knowledge of the extrinsic control of cancer cell dormancy has implications for understanding cancer pathogenesis and the potential to unlock opportunities for developing innovative treatment strategies.

## Methods

### Mice and *in vivo* mouse models

Six-to-eight-week-old C57BL/KaLwRijHsd (BKAL) male mice (Harlan, Netherlands) were injected intravenously with 2 × 10^6^ syngeneic 5TGM1-eGFP (a gift from Dr Oyajobi, University of Texas Health Sciences Center San Antonio, Texas, USA) or 5T33MM murine myeloma cells in 200 μl PBS^54^,^55^. Cells were negative for mycoplasma. On specified days post injection, mice were killed for FACS analysis and two-photon imaging of explanted bones. In separate studies, 6–8-week-old male BKAL mice were injected with 5T2MM murine myeloma cells and killed 7 weeks later to examine tumour colony formation. BKAL mice were also crossed with mice expressing emerald GFP under the type-I collagen promoter (pOBCol2.3GFPemd)^38^ to produce C57BL/KaLwRijHsd.Cg-Tg (Col1a1-GFP) denoted as Kal-Col-GFP mice to mark osteoblastic cells *in vivo*. For drug studies, BKAL mice were injected with DiD-labelled 5TGM1-eGFP cells. After 2 weeks, mice were injected, intraperitoneally, three times a week for 2 weeks with melphalan (Sigma-Aldrich, 5 mg kg^−1^ in 17.5% ethanol) or vehicle^56^; or twice a week, subcutaneously, with bortezomib (Sigma-Aldrich, 0.7 mg kg^−1^ in 0.005% dimethylsulphoxide). Mice were killed 4 weeks later and 1 or 2 weeks after cessation of melphalan treatment. Tibia, femurs and spleens were isolated for FACS and two-photon imaging. Drug studies were performed in two separate experiments. Recombinant soluble RANKL-GST fusion protein (RANKL, Oriental Yeast Company Ltd, Tokyo, Japan) or saline was administered at a dose of 0.5 mg kg^−1^ intraperitoneally daily for 3 days in naive mice before harvest on day 4. RANKL was administered on days 4–6 post cell injection and experiments ceased 18 days later. Experiments were approved by the Garvan Institute/St Vincent's Hospital (#12_20), University of Sheffield (PPL 40/3,462) and Vrije Universiteit Brussel (#13-281-5) Animal Ethics Committees.

### Cancer cell lines and intravital labelling of dormant cells

5TGM1-eGFP and 5T33MM myeloma cells were cultured in RPMI-1640 supplemented with 10% fetal calf serum (FCS) and 1% penicillin–streptomycin, and incubated in 5% CO_2_ at 37 °C (refs 54, 55). 5T2MM cells were maintained by serial passage in BKAL mice^54^. Cells were labelled with DiD, or 3H-Indolium, 5-[[[4-(chloromethyl)benzoyl]amino]methyl]-2-[3-(1,3-dihydro-3,3-dimethyl-1-octadecyl-2H-indol-2-ylidene)-1-propenyl]-3,3-dimethyl-1-octadecyl-, chloride (CMDiI) (Molecular Probes).

### *In vitro* conditioned media and co-culture experiments

Primary osteoblasts were isolated from calvaria of 2–4-day-old C57BL/6 mice and differentiated in MEMα supplemented with ascorbic acid, β-glycerol phosphate, ribonucleosides and 10% FCS for 4 weeks^57^,^58^. DiD-labelled 5TGM1-eGFP cells were cultured either in media, or in media conditioned with 5TGM1, RAW264 cells or primary osteoblasts. DiD-labelled 5TGM1-eGFP cells were co-cultured with MC3T3 murine osteoblast-like cells plated in RPMI, supplemented with 10% FCS, 1% penicillin/streptomycin and 1% Glutamax.

### Two-photon microscopy of explanted tibias and femurs

At the University of Sheffield, the tibia or femur were embedded in OCT solution (Tissue-Tek) and snap-frozen in liquid nitrogen before cryosectioning. Images were acquired with an inverted Zeiss 510 META (Carl Zeiss). Two-photon excitation at 820 nm was achieved with a Chameleon XR Ti:Sa laser (Coherent) and fluorescence detected using the following: BP435-485 to detect blue (second-harmonic generation), BP 500–550 to detect green (GFP), BP 565–615 IR to detect orange/red (CMDiI) and BP 650–710 nm to detect far-red (DiD). In some images (Figs 3 and 4; Supplementary Fig. 2) this was pseudocoloured white. Three-dimensional (3D) mosaic tiles were performed at 128 × 128 pixel resolution and individual 450 × 450 × 150 μm volumes imaged at 256 × 256 pixel resolution in 2-μm z-steps. Three-dimensional volumes were reconstructed and analysed using Volocity software (Improvision, Perkin Elmer). At the Garvan Institute of Medical Research, imaging was performed on intact tibias or cryosectioned femurs using an upright Zeiss 7MP microscope (Carl Zeiss). We used LBF 760 and BSMP2 760 filters to enable detection of the far-red emission of BSA-Alexa Fluor680 and DiD. Four external non-descanned detectors were used to detect blue (second-harmonic generation, SP 485), green (BP 500–550), red (BP 565–610) and far-red (BP 640–710). Infrared excitation at 920 nm was provided by a Chameleon Vision II Ti:Sa laser (Coherent Scientific). Each 512 × 512-pixel *xy*-plane was acquired at 2–3-μm z-step intervals. Time-lapse images were acquired at 30-s intervals. Images were acquired using ZEN 2009 software (Zeiss) and raw image files processed using Imaris (Bitplane). Red dormant cells and green activated cancer cells were counted using the spot detection function in Imaris.

### Real-time intravital two-photon microscopy of the tibia

Mice were anaesthetized as described^59^. Anaesthetized mice were kept warm using a customized heated SmartStage (Biotherm). An incision was made over the medial surface of the tibia and soft tissue resected. Mice were intravenously injected with a single dose of Alexa Fluor680/bovine serum albumin (2.5–10.0 mg kg^−1^, Invitrogen) to label blood vessels. 5TGM1-eGFP cells were injected four frames (2 min) after commencement of imaging. A Gaussian filter was applied and the display adjusted to reduce background noise and improve contrast in Imaris. Videos were compiled and annotated using Adobe AfterEffects (Adobe). Myeloma cells were counted as circulating cells if they entered and left the imaging volume within two consecutive frames. Colonizing cells entered, crawled and arrested within the imaging volume and could be found in the same location again at least 5 min later.

### Longitudinal intravital two-photon microscopy of the tibia

For longitudinal imaging, 2 × 10^6^ DiD-labelled 5TGM1-eGFP cells were injected 7 days before the initial imaging session. During imaging, a 3D mosaic tile image was captured to include the growth plate and other landmarks as fiducial markers to enable repeated imaging of the same volume. Mice were recovered after imaging, the skin sutured and buprenorphine hydrochloride 0.5 mg kg^−1^ subcutaneously administered. Mice were re-imaged on days 14 and 21 in the same region based on the position of the fiducial markers.

### Micro-computed tomography of the tibia

Bones were scanned using a μCT scanner (Model 1172; Skyscan) at 50 kV, 200 mA with a 0.5-mm aluminium filter using a pixel size of 4.3 μm. Images were captured every 0.4̊ through 180̊, reconstructed and analysed using NRecon and CTAn software (Skyscan). Three-dimensional models were created using the Drishti-2 tool (https://code.google.com/p/drishti-2/).

### Histology

Bones were fixed in 4% paraformaldehyde, decalcified in EDTA, embedded in paraffin and 4-μm sections were cut. Antigen retrieval was performed in trypsin (Biocare Medical) at 37°C for 10 min, and washed for 5 min in PBS. Nonspecific binding was blocked in 10% FCS and 10% normal goat serum (S-1000, Vector Laboratories) for 1 h at room temperature (RT). Primary antibody (rat anti-mouse anti-CD138, clone 281-2, BD Pharmingen) and isotype control (purified rat IgG2a, κ-isotype control, clone R35-95 BD Pharmingen) were incubated for 1 h at RT at a dilution of 1:300 and washed in PBS. Endogenous peroxidase activity was quenched with 3% hydrogen peroxide (Sigma) for 30 min at RT. Primary antibody was detected with a secondary biotinylated goat anti-rat IgG F(ab)_2_ fragment (SC-3,826, Santa Cruz) incubated for 30 min at RT and washed in PBS before incubation with strepavidin-horseradish peroxidase (DakoCytomation) for 30 min at RT. Antigen/antibody complex was visualized with 3,3,diaminobenzadine (SK-4105, Vector Laboratories) and counter-stained with haematoxylin. CD138^+ve^ cells and colonies were counted and data expressed/bone section. Identical data were obtained when data were expressed per unit area.

### FACS analysis

Bones were flushed with PBS/2% FCS, cut longitudinally and the inner surface was scraped with a scalpel blade. Bones (including the epiphysis) were minced and fragments flushed with PBS/2% FCS. Cells were syringed five times through a 23-G needle and filtered through a 100-mm nylon cell strainer (Nunc). Red cells were lysed with 0.15 M NH4Cl. Cells were resuspended in PBS/2% FCS and filtered through a 70-mm strainer. For Ki67 staining, samples were incubated with Fixable Viability Dye eFluor780 (eBioscience) and then Fc-blocked with anti-mouse CD16/32 before fixation with the Nuclear Factor Fixation and Permeabilization Buffer Set (BioLegend). Cells were then incubated with PE anti-mouse Ki67 at a dilution of 1:100 (BioLegend) and DAPI to stain DNA. For CD11b, Gr-1, CD138 and B220 staining, samples were incubated with Fixable Viability Dye eFluor780, then Fc-blocked with anti-mouse CD16/32. Cells were then incubated with PE anti-mouse B220, PERCP-Cy5.5 anti-mouse CD11b, Pe-Cy7 anti-mouse CD138 (clone 281-2) and Brilliant Violet 450 anti-mouse Gr-1 (BioLegend) at a dilution of 1:100. Samples were acquired on a FACSCanto II or LSR II SORP (BD Biosciences). Analysis was performed with FlowJo software (Tree Star). For microarray analysis, up to 50,000 cells were sorted using BD FACSAria II or BD FACSAria III (BD Biosciences) directly into 0.5 ml of TRIzol LS reagent (Life Technologies Australia).

### Transcript profiling

Total RNA was extracted using Trizol LS reagent and its integrity measured using a Total RNA Pico Chip (Agilent Technologies) according to manufacturer's protocol. Complementary DNA was prepared and amplified using the NuGEN Ovation Pico System V2, and hybridized with GeneChip Mouse Gene 2.0 ST Arrays (Affymetrix). Normalization and probe set summarization was performed using the robust multichip average implemented through the Affymetrix library within R^60^. Control probe sets were removed leaving 31,235 probe sets on the array following batch-correction using SVA package in R^61^. Differential gene expression was assessed for each probe set using an empirical Bayes, moderated *t*-statistic implemented in Limma using the limmaGP tool in GenePattern^60^. GSEA^34^ was performed with the GenePattern tool GSEA pre-ranked using a ranked list of the Limma-moderated *t*-statistics against version 3.0 of the ‘C2_all' curated gene sets from the MSigDB supplemented with gene lists related to stem cell regulation, metastasis and melphalan treatment^62^,^33^. Functional annotation of 867 significant genes (fold change >2, *Q*-value<0.05) was performed using database for annotation, visualization and integrated discovery (DAVID)^64^,^65^. All analyses were performed using GenePattern software^66^ and are available at http://pwbc.garvan.unsw.edu.au/gp/. The microarray data have been deposited in the GEO database under accession code GSE57695.

### Patient serum analysis

Newly diagnosed myeloma patients, who had not received chemotherapy, were included in this study. Ethical approval for this study was obtained from the Royal Adelaide Hospital Human Research Ethics Committee and informed consent was obtained in accordance with the Declaration of Helsinki. Staging was based on the ISS criteria. Of the 118 patients examined, 53 were female (median age at diagnosis, 67 years; range, 47–83 years) and 66 were male (median age at diagnosis, 61 years; range, 49–74 years). Serum β2m, a marker of tumour burden in renal-sufficient patients, creatinine concentrations and creatinine-based renal parameters (estimated glomerular filtration rate and creatinine clearance) were evaluated using an Olympus AU5400 Chemistry Analyzer. Patients with renal insufficiency were excluded from further analysis. Serum levels of C-terminal collagen crosslinks (CTX-1) were measured using a Roche modular E170 analyzer (Roche Diagnostics, Castle Hill, Australia).

### Statistical analysis

Data were analysed using Prism software (GraphPad). One-way analysis of variance (ANOVA) and multiple comparisons were performed using Tukey's correction. Unpaired *t*-tests were performed when comparing two populations. Non-parametric data were assessed using Kruskal–Wallis one-way ANOVA. Two-way ANOVA were used for grouped analyses (co-culture) with Sidak's correction. Correlations between serum CTX-1 and β2m were assessed using linear regression analysis. All data are expressed with error bars representing s.e.m.

## Additional information

**Accession codes**: The microarray data have been deposited in the GEO database under accession code GSE57695.

**How to cite this article:** Lawson, M. A. *et al*. Osteoclasts control reactivation of dormant myeloma cells by remodelling the endosteal niche. *Nat. Commun.* 6:8983 doi: 10.1038/ncomms9983 (2015).

## Supplementary Material

Supplementary Information Supplementary Figures 1-2 and Supplementary Tables 1-2

Supplementary Movie 1 Tumor cells circulate through bone. Time-lapse images from real-time intravital two-photon microscopy demonstrating a tumor cell circulating through bone at 1 hour post cell injection. 5TGM1-eGFP cell, green; vasculature, red; bone SHG, blue. Left screen lateral xy plane, right screen axial xz plane. Timestamp (hh:mm:ss) starts at 00:00:00 when cells are injected into the tail vein. Scale bar, 20μm. Data is representative of 11 experiments.

Supplementary Movie 2 Tumor cells colonize bone. Time-lapse images from real-time. intravital two-photon microscopy demonstrating a tumour cell colonising bone within 9 minutes of cell injection. 5TGM1-eGFP cell, green;vasculature, red; bone SHG, blue. Left screen lateral xy plane, right screen axial xz plane. Timestamp (hh:mm:ss) starts at 00:00:00 when cells are injected into the tail vein. Scale bar, 20μm. Data is representative of 11 experiments.

Supplementary Movie 3 GFP+ DiD+ cells persist adjacent to bone surfaces. Serial zstacks from two-photon imaging of explanted femur 28 days after cell injection. GFP+ DiD+ cells localize to bone surfaces. Bone SHG, blue; GFP+ DiDneg cell, green; GFP+ DiD+ cells, yellow (red/green). Scale bar, 40μm. Data represents at least 10 experiments.

Supplementary Movie 4 GFP+ colonies expand overtime. Maximum intensity projection image in the x, y and z planes of a zstack of the same GFP+ tumor colony at 14 and 20 days imaged using our intravital technique. Bone SHG, blue; GFP+ DiDneg cell, green; GFP+ DiD+ cells, yellow (red/green).
