# Supplementary material for: Osteoclasts control reactivation of dormant myeloma cells by remodelling the endosteal niche
Source: Nat Commun. 2015 Dec 3;6:8983. doi: 10.1038/ncomms9983 (PMC4686867; doi:10.1038/ncomms9983)
Supplement: Supplementary Information — Supplementary Figures 1-2 and Supplementary Tables 1-2 [file ncomms9983-s1.pdf]

Supplementary Figure 1

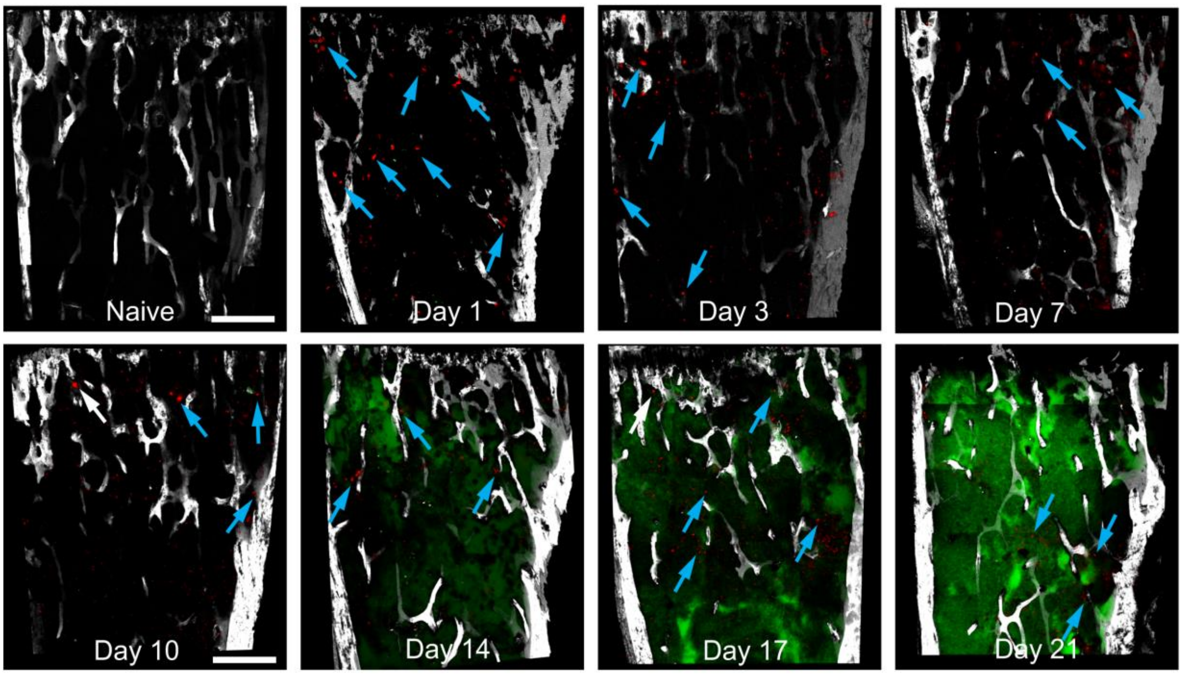

Supplementary Figure 1. Dormant cells persist *in vivo* 21 days post injection. Mosaic tile images from two-photon imaging of explanted tibia up to 21 days following injection of 5TGM1 a cells compared to naïve mice not injected with 5TGM1 cells (first panel). $GFP^{+}DiD^{+}$ cells (blue arrows) located adjacent to bone surfaces at all time points up to 21 days post injection. Scale bar, 200µm. Bone SHG, white; $GFP^{+}DiD^{+}$ cells, red; $GFP^{+}DiD^{neg}$ cells, green. Data represent at least 3 experiments.

Supplementary Figure 2.

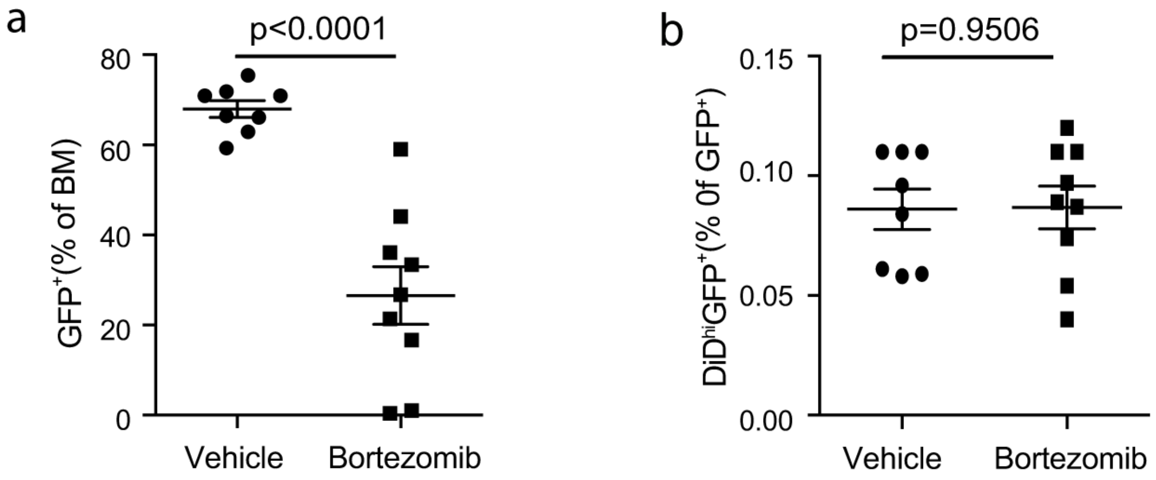

Supplementary Figure 2. Proliferating and dormant cells populations respond similarly to the proteasome inhibitor Bortezomib. FACS analysis of bone marrow from vehicle and Bortezomib treated 5TGM1 burdened mice. **a)** GFP^{+} cells (% of bone marrow cells) and **b)** $GFP^{+}DiD^{hi}$ cells (% of GFP^{+} cells). Data show mean ± SEM and represent 8-10 mice per group, unpaired ttest.

Supplementary Table 1:
Gene ontologies associated with cell cycle that are down-regulated in dormant (DiD^{hi}) cells

Cell Cycle Process	Gene Ontology:ID	Direction	Genes
Cell division	GO:0051301	Down	*2610039C10Rik, 2610002M06Rik, Anapc10, Anln, Cenph, F630043A04Rik, Fam83d, Katna1, Kif20b, Ndc80, Nuf2, Nup37, Pmf1, Ska1, Smc2, Vps4a, Wee1*
Mitotic cell cycle	GO:0000278	Down	*2610039C10Rik, Anapc10, Anln, Cenph, F630043A04Rik, Fam83d, Hinfp, Katna1, Kif20b, Ndc80, Nuf2, Nup37, Pmf1, Rps6ka2, Ska1, Smc2, Wee1*
Cell cycle process	GO:0022402	Down	*2610039C10Rik, Anapc10, Anln, Cenph, F630043A04Rik, Fam83d, Hinfp, Katna1, Kif20b, Klhdc3, Mlh3, Ndc80, Nuf2, Nup37, Pmf1, Rad50, Rps6ka2, Ska1, Smc2, Suv39h2, Trip13, Wee1*

All enrichment categories Bonferroni <0.05.

Supplementary Table 1. Gene ontologies associated with cell cycle that are down-regulated in dormant (DiD^{hi}) cells.

Supplementary Table 2: Differentially expressed genes in dormant (DiD^{hi}) cells

Top 100 up regulated in dormant (DiD^{hi}) cells

Gene Symbol / Transcript ID	absFC	Gene Symbol / Transcript ID	absFC	Gene Symbol / Transcript ID	absFC
LOC100862132	13.11	Mir302b	5.66	ENSMUST00000157188	4.24
Lilrb4	10.82	Bcl2a1a	5.59	ENSMUST00000158455	4.19
Ctsc	10.04	Cd97	5.51	ENSMUST00000103327	4.17
Fabp5	9.90	H2-Aa	5.44	Bcl2a1b	4.15
Anpep	9.60	AF251705	5.43	Irf7	4.14
Mpeg1	9.32	Lilrb3	5.33	Ifi205	4.12
Spic	8.74	Gm19528	5.32	Fyb	4.11
Gm9340	8.21	Snord37	5.31	Ly6g	4.09
C1qb	8.12	Ccl12	5.20	ENSMUST00000158348	4.09
ENSMUST00000158834	7.96	ENSMUST00000158950	5.18	Atp6v0d2	4.08
Fcgr1	7.91	Gm20559	4.97	Gpnmb	4.07
Fcer1g	7.89	S100a6	4.96	Lcp2	4.07
Arl6ip1	7.86	ENSMUST00000104369	4.90	AW112010	4.07
Tyrobp	7.78	Snord35b	4.90	LOC100862211	4.05
ENSMUST00000158671	7.30	Tlr7	4.84	Axl	4.02
Tmem106a	6.94	DQ340292	4.82	ENSMUST00000083119	4.02
Ms4a7	6.83	ENSMUST00000158532	4.81	Cxcl9	3.98
Cd63	6.79	Sirpa	4.76	Hmox1	3.97
Mmp12	6.77	Ccl8	4.73	Hpgd	3.94
H2-Ab1	6.75	Cd84	4.64	Cyp4v3	3.93
Vcam1	6.73	Rgs2	4.61	Lyz2	3.92
Lst1	6.69	Fabp4	4.56	Vim	3.88
AB124611	6.67	Sh3bgrl3	4.54	ENSMUST00000160463	3.87
Gm11428	6.46	Fam105a	4.47	Olfr739	3.87
Ifitm2	6.39	Ccl2	4.46	Rgs1	3.83
Bcl2a1d	6.17	Emp1	4.43	Npl	3.80
Slc40a1	6.09	Ngp	4.39	Cd180	3.80
ENSMUST00000082533	6.02	Ctsh	4.37	Mir302c	3.78
C4b	5.89	Cd5l	4.34	Slc15a3	3.75
Rtp4	5.82	Lgals3bp	4.33	ENSMUST00000173249	3.75
Clec4a3	5.76	Itgax	4.33	Mfsd1	3.73
Pltp	5.76	ENSMUST00000083425	4.30	Ifi44	3.71
Fcgr4	5.68	Csf1r	4.30	Mrc1	3.70
				Gpr65	3.68

Top 100 down regulated in dormant (DiD^{hi}) cells

Gene Symbol / Transcript ID	absFC	Gene Symbol / Transcript ID	absFC	Gene Symbol / Transcript ID	absFC
ENSMUST00000082919	9.06	Klf12	3.26	Ankrd49	2.91
Snora17	8.37	Slc30a6	3.26	ENSMUST00000157749	2.90
4930522L14Rik	6.07	ENSMUST00000157817	3.24	2610039C10Rik	2.89
ENSMUST00000158766	4.94	Pip4k2b	3.24	ENSMUST00000175498	2.89
Ppan	4.90	Zfp433	3.24	Fam58b	2.87
Snord73a	4.90	Zxdb	3.21	Esco2	2.87
5730408K05Rik	4.89	Gm9958	3.17	Zfp825	2.86
1810007I06Rik	4.69	Hist1h2bh	3.17	Nts	2.85
Gm15363	4.56	ENSMUST00000083721	3.15	Tmed1	2.85
Gm5751	4.51	ENSMUST00000158504	3.15	ENSMUST00000104118	2.85
ENSMUST00000122729	4.49	ENSMUST00000083026	3.14	Ppp6r2	2.84
ENSMUST00000083102	4.40	Ndc80	3.14	ENSMUST00000175017	2.84
Trip13	4.03	Ndufb11	3.13	1810031K17Rik	2.83
Mir15a	4.02	Hist1h1a	3.13	Bckdhb	2.83
Copz1	3.89	2310058D17Rik	3.10	17475704	2.82
Hist1h2bg	3.83	ENSMUST00000102427	3.09	Lmf2	2.82
ENSMUST00000157558	3.81	ENSMUST00000083993	3.09	Fam20b	2.81
Mir103-2	3.72	Hmga1-rs1	3.08	BC080809	2.81
Mettl21d	3.71	ENSMUST00000122495	3.07	ENSMUST00000158552	2.79
Odf2l	3.68	ENSMUST00000082493	3.05	ENSMUST00000175310	2.79
ENSMUST00000157098	3.63	9130206I24Rik	3.04	Mir5099	2.79
Snora69	3.62	ENSMUST00000054878	3.03	ENSMUST00000173128	2.78
ENSMUST00000157760	3.59	2210418O10Rik	3.01	ENSMUST00000157125	2.78
E330020D12Rik	3.58	AJ555622	3.01	ENSMUST00000083107	2.77
ENSMUST00000082596	3.42	ENSMUST00000157643	2.98	Stk3	2.76
Jrkl	3.39	ENSMUST00000157653	2.97	Atg5	2.75
2410019O14Rik	3.37	Rnf7	2.97	Tmem55a	2.74
Snord15b	3.37	Ddit4	2.93	Taf8	2.73
Gm20186	3.36	LOC100861983	2.93	ENSMUST00000158372	2.73
Hrsp12	3.35	Sptlc1	2.92	LOC100862646	2.73
4932425I24Rik	3.31	Snord11	2.92	2610044O15Rik	2.71
Mrpl34	3.29	1190007I07Rik	2.92	Qtrtd1	2.71
Pmf1	3.27	Gstt2	2.91	Mzt2	2.71
				Pmaip1	2.71

All genes Q value <0.05.

Supplementary Table 2. Top 200 differentially expressed genes in dormant (GFP^+DiD^{hi}) cells.

Gene lists based on filtering criteria fold change >2, Q value <0.05.
